# Investigation of the Digestibility, Fecal Characteristics, and Palatability of Oil Mil By-Products as a Plant-Based Protein Source in Canine Diets

**DOI:** 10.3390/ani15223279

**Published:** 2025-11-13

**Authors:** Ammelie Godglück, Julia Hankel, Volker Wilke, Cristina Ullrich, Christian Visscher

**Affiliations:** Institute for Animal Nutrition, University of Veterinary Medicine Hannover, Foundation, Bischofsholer Damm 15, D-30173 Hannover, Germany; julia.hankel@tiho-hannover.de (J.H.); wilke@tierklinik-sarstedt.de (V.W.); cristina.ullrich@tiho-hannover.de (C.U.); christian.visscher@tiho-hannover.de (C.V.)

**Keywords:** dogs, plant-based protein, digestibility, fecal characteristics, palatability

## Abstract

This study investigates the digestibility, fecal characteristics, and palatability of plant-based protein sources in canine diets. With an increasing number of pet owners adopting vegan lifestyles for ethical, health, and environmental reasons, the demand for plant-based dog diets has risen. Using plant by-products can also be a practical and sustainable option for feeding pets. This study included eight healthy female dogs fed four different plant-based diets using a Latin square (4 × 4) crossover design, with linseed press cake, sunflower press cake, pumpkin press cake, and soy press cake as protein sources. To measure the acceptance of the protein sources, a five-bowl test was used. The apparent total tract digestibility of the different diets and the fecal quality were compared as well as the acceptability of the individual protein sources. The results showed significantly (*p* < 0.0001) better acceptance for the pumpkin press cake compared to the other three tested press cakes, whereas the addition of potato flakes changed the acceptance and showed no significant (*p* = 0.1105) differences between the press cakes. Among the tested diets, the highest protein digestibility was observed for the pumpkin diet (85.11% ± 1.77%), followed by the sunflower (84.23% ± 2.20%) and soy diets (82.35% ± 3.37%), while the linseed diet showed the lowest protein digestibility (77.52% ± 6.04%).

## Simple Summary

This study investigated how well dogs can utilize different plant protein sources, how these diets affect fecal quality, and how readily the dogs accept them. Eight healthy dogs were fed four diets containing different protein sources: pumpkin press cake, sunflower press cake, linseed press cake, and soy press cake. Acceptance was evaluated using a multiple-bowl test. The results showed a clear preference for pumpkin press cake compared to the other protein sources. However, when potato flakes were added to the diets, no significant differences in acceptance were observed. Regarding protein digestibility, the pumpkin diet achieved the highest values, followed by sunflower and soy, whereas linseed showed the lowest digestibility. Fecal quality also varied depending on the protein source. These findings provide valuable insights into the potential of plant by-products as sustainable and digestible protein sources in canine nutrition and support the development of balanced plant-based dog foods.

## 1. Introduction

Since their domestication over the last 13,000 to 17,000 years, dogs have increasingly adapted to the eating habits of humans and evolved from being primarily carnivorous to becoming more and more omnivorous [1]. The increased copies of the AMY2B gene, which is responsible for the digestion of starch, compared to the wolf and dingo also suggest that the modern dog has adapted to an omnivorous diet [2]. In addition, it was shown that, historically, dogs from regions where agriculture is advanced, like Europe or Asia, have significantly higher copy numbers of the AMY2B gene than dogs from regions with less agricultural influence, like Arctic regions, suggesting that they may have adapted to the dietary habits of humans [3]. In early Neolithic agricultural societies in China, coprolite analysis reveals that dogs consumed both plant- and animal-based foods in various proportions, reflecting a close commensal relationship with humans and suggesting that an omnivorous diet has deep evolutionary roots [4]. Humans and dogs have co-evolved for thousands of years, and the dietary trends of these two species have adapted as a result [5].

Many different factors influence the choice of food that people choose for their dogs; in particular, geographic differences seem to play an important role [6]. In recent years, people have become increasingly interested in vegan and vegetarian diets; the most searched for diets by Google users were veganism (19.54%) and vegetarianism (15.09%). In fact, only in Canada was a gluten-free diet and in Brazil and Scandinavia a low-carbohydrate diet more popular [7]. A growing number of people have adopted a vegetarian or vegan lifestyle in the past few years for ethical, health, and environmental reasons [8]. Certain pet owners care about all animals, including livestock, and the condition of the environment, and live vegan for these reasons [9]. Many of these pet owners find themselves in an ethical and moral conflict when feeding their pets food with animal-derived ingredients [9]. However, over 70% of vegan pet owners surveyed would feed their dog a plant-based diet if one were available that met their criteria for passing on the benefits of a plant-based diet to their pets, in an effort to promote their well-being and health in line with their own ethical and nutritional principles [10]. In one study where 2639 dog owners in the United Kingdom were asked how they fed their dogs, 12% fed a vegan diet, while 55% fed a conventional diet, and 33% fed raw meat [11]. In a second study, that reported on the dietary information of 2940 dogs that mainly came from the United States and the United Kingdom, 97% of the dogs were fed a diet that contained meat and 10.4% of the dogs were intermittently fed vegetarian or plant-based dog food [10].

From an additional perspective, dogs possess a noteworthy ecological footprint that should not be underestimated. A study could show that dogs and cats in the USA consume as much dietary energy as 62 million Americans [12]. The main part of a dog’s emissions is caused by the food, including production, packing, and transportation; the second part is caused by the dog’s output, including direct emissions like feces and urine and indirect emissions like plastic bags for disposal and the cleaning of the streets [13]. However, when comparing a vegetarian diet with a meat-based diet, the nitrogen content in the feces was slightly lower in the vegetarian diet [14]. Also, premium dog food contributed 2.3 times the emission intensity compared to the market-leading dog food due to the very high meat content, which results in higher greenhouse gas emissions and agricultural land use for these products [15].

Therefore, from an environmental point of view, it may make sense to use alternative protein sources in dog diets [16]. Previous studies have shown that adult dogs can be maintained on a balanced plant-based diet for up to three years without adverse health effects, and some findings even suggest potential health benefits [17,18]. For example, data from a large-scale survey of 1189 dog owners in Canada and North America showed that owners who fed their dogs a vegan diet reported significantly fewer cases of gastrointestinal and renal diseases and even a significantly longer life expectancy of their dogs than those dog owners who fed a meat-based diet [19]. However, in another survey of 2536 dog owners, the number of self-reported veterinary visits was reported and compared among three different main diets: conventional meat, raw meat, and vegan; the results showed that 60% of vegan dogs had one or no veterinary visit, while 49% of dogs fed a conventional meat diet had one or no veterinary visit [20,21]. Nonetheless, this type of owner-reported data does not allow for conclusions regarding overall health status or long-term effects. In a trial where dogs were fed a plant-based diet for 12 weeks and had a physical examination, an echocardiogram, and a blood test afterwards, median hematologic and biochemical values were within normal limits, and in the echocardiographic data, there was no statistical difference between the plant-based group and the control group [17,22].

As adult dogs are able to synthesize the amino sulfone acid taurine themselves from sulfur-containing amino acids, it is therefore advisable to adhere to the recommendations for the supply of sulfur-containing amino acids [22]. While dogs need energy and essential nutrients, they do not inherently require animal components per se [23]. Moreover, there are dogs that suffer from food allergies or a food intolerance, which is mainly caused by animal proteins; for these dogs, alternative protein sources can be a good option [24].

Plant-derived press cakes, the solid residues remaining after oil extraction from seeds or nuts, exhibit substantial variation in their nutrient composition, particularly regarding crude protein and fiber contents [25]. For instance, press cakes from soy, rapeseed or sunflower are typically characterized by high protein concentrations and favorable amino acid profiles, rendering them valuable as alternative protein sources in feed or food formulations [26]. Press cakes are already utilized in livestock nutrition as sustainable feed ingredients, providing valuable protein and energy [27].

Based on the arguments presented, it seems reasonable to investigate the use of new vegetable protein sources for dogs to be able to make further statements about the applicability and digestibility of vegetable protein. Therefore, the objective of this study was to compare the palatability (hemp press cake, linseed press cake, sunflower press cake, rapeseed press cake, and pumpkin press cake), fecal quality, and apparent nutrient digestibility of different oil mill by-products as plant-based protein sources (sunflower press cake, linseed press cake, pumpkin press cake, and soy press cake).

## 2. Materials and Methods

### 2.1. Animals and Housing

The study was conducted at the Institute for Animal Nutrition, University of Veterinary Medicine Hannover, Foundation, Hannover, Germany. The study included eight healthy female beagle dogs owned by the Institute for Animal Nutrition at the University of Veterinary Medicine Hannover, where they are housed and used for feeding trials. The animals had a median age of 3.37 ± 1.3 years and a mean body weight (BW) of 9.17 ± 0.40 kg at the start of the study. The dogs’ health status was checked before the beginning of the experiment by clinical examination, and they were regularly vaccinated and dewormed. The body condition score (BCS) was 5 ± 0.32 of a total of 9 according to Laflamme during the entire experiment [28]. All experimental dogs were managed and handled following the guidelines approved by the animal welfare officers of the University of Veterinary Medicine Hannover, Foundation, Hannover, Germany (TVO-2023-V-39).

### 2.2. Palatability Testing

The acceptance of the protein sources was tested in a five-bowl test modified from the four-bowl test by Curso-Almeida [29]. The dogs were led to a food bar with five identical bowls. The bowls were at a uniform distance from each other. First, the dogs were led past the bowls once and were able to sniff the contents but were not allowed to eat. Then the dogs were unleashed and had 10 min to eat, during which time they were alone in the kennel without human supervision. The first choice was judged from outside the kennel. Each dog was given freshly cleaned bowls, which were cleaned daily with the same detergent in the same dishwasher. The five-bowl test was conducted every morning before the dogs were given their usual food. The first choice was documented to determine acceptance. Feed intake was determined by weighing the left-over feed after feeding. To achieve a uniform consistency, all rations were soaked in water. The position of the bowls was changed daily to eliminate any potential bias.

The three protein sources that showed the highest acceptance and most favorable chemical characteristics were selected for the formulation of the diet used in the apparent digestibility trials. Soy press cake was included as a control.

### 2.3. Apparent Digestibility

The apparent digestibility trial was conducted with eight dogs (*n* = 8) using a 4 × 4 Latin square crossover design. The adaptation period for habituation to the diet took 9 days, followed by a 5-day period of fecal collection for nutrient digestibility, which fulfills the minimum requirement of four days [30]. Between experimental periods, a 2-week washout phase was implemented during which all dogs were fed a standard diet to minimize carryover effects between dietary treatments. A total fecal collection was performed, and all feces excreted during the collection phase were stored at −20 °C until analysis. Feed intake was recorded daily; all dogs consumed their entire meals within 10 min after feeding. The dogs were fed once per day at 07:00 and received water ad libitum.

### 2.4. Diet and Feeding

The palatability testing included two trials. In the first run, the oil mill by-products were offered in isolation (50 g press cake in comparable dry matter and 50 g water per bowl); in the second run, the oil mill by-products were offered with increasing proportions of potato flakes (Table 1). The oil mill by-products on offer were hemp press cake, linseed press cake, sunflower press cake, rapeseed press cake, and pumpkin press cake (Table 2).

For the digestibility study, the three protein sources with the best results in palatability testing and the most comparable nutrient composition were selected. In addition, soy press cake was added as a control diet. During the digestibility study, each dog was given four different plant-based diets, each with a different protein source. The diets consisted mainly of four different oil mill by-products as protein source, potato flakes, potato protein, and linseed oil (Table 3). The diets were extruded dry food with a complementary feed on top (Table 4). The diets were isonitrogenous and were calculated to be isoenergetic and were designed according to FEDIAF recommendations [30]. To improve the acceptance of the complementary feed, 50 mL of water was added to each ration. The daily food ration for each dog was calculated by using the equation for the daily energy requirements of adult dogs (0.42 MJ metabolizable energy BWˆ0.75/d).

### 2.5. Chemical Analysis

The press cakes used in the palatability testing were analyzed for moisture, crude ash, crude protein, crude fiber, crude fat, calcium, phosphorus, and amino acids. Diets were analyzed for moisture, crude ash, crude protein, crude fiber, insoluble dietary fiber, crude fat, calcium, phosphorus, and amino acids. Feces were analyzed for moisture, crude ash, crude protein, crude fat, crude fiber, and amino acids. All analyses were conducted according to the methods of the VDLUFA [31]. Each determination was performed in duplicate (*n* = 2) to ensure analytical reliability, and recovery rates were verified using certified reference materials to confirm method accuracy. The moisture content was determined by drying at 103 °C until weight constancy. The crude ash was analyzed by incineration in the muffle furnace for six hours at 600 °C. Crude protein content was determined by the Dumas combustion method using the Vario max CNS (Elementar Analysesysteme GmbH, Langenselbold, Germany). The crude fiber content was determined after washing in dilute acids and alkalis, while the crude fat was determined using automatic hydrolysis and extraction with Hydrotherm and SOXTHERM 416 (Soxtherm 416/406, C. Gerhardt GmbH & Co. KG, Königswinter, Germany). The procedure for determining insoluble dietary fiber involved sequential enzymatic digestion of 1 g of dried food sample using heat-stable α-amylase, protease, and amyloglucosidase. Following the removal of insoluble components, soluble fiber was precipitated from the filtrate by adding distilled water and ethanol. To determine the TDF, the residue was filtered, dried, and weighed. The TDF content was corrected for protein and ash content. The mineral content was measured by atomic absorption spectrometry (Unicam Solaar 116, Thermo Fisher Scientific GmbH, Dreieich, Germany). Two digestions were carried out for the amino acids, a hydrolysis and an oxidation for the sulfur-containing amino acids. Amino acid content was analyzed by ion-exchange chromatography (Biochrom Bio 30+, Biochrom Ltd., Cambridge, England). Tryptophan content was measured using HPLC (Fluorescence Detector RF-10A XL, Shimadzu GmbH, Duisburg, Germany) after digestion for 24 h with a lithium hydroxide solution and adjustment of the solution to pH 3.

The collected material was weighed every evening, and the dry matter content was determined from a subsample of 10% of the feces. The remaining fecal samples were stored at −20 °C until the end of the trial. The five-day fecal samples from each dog were thawed, mixed, and homogenized [14]. To calculate the gross and metabolic energy, the following formulas were used [32]:

GE (MJ/100 g) = 0.02385 CP + 0.03934 EE + 0.01717 CF + 0.01717 NFE

DE (MJ/100 g) = GE (MJ/100 g) × VGE (%)/100

ME (MJ/100 g) = DE (MJ/100 g) − 0.00434 MJ × CP (g/100 g)

VGE (%) = 91.2 − 1.43 CF (%)

To calculate the apparent digestibility, the following formula was used [32]:

Apparent digestibility (%) = (food − feces)/food × 100

### 2.6. Fecal Quality

The fecal quality was determined every day during the collection phase. Therefore, the number of defecations was counted, and two fecal scoring systems were used to capture the consistency and the fecal shape. To score the consistency, the Waltham fecal scoring system was used. This system uses a 5-point scale (1 = “bullet like”, crumbles with little pressure; 2 = well formed, does not leave a mark when picked up; 3 = moist, beginning to lose form, leaving a definite mark when picked up; 4 = most or all form is lost, no real shape, 5 = completely liquid stool). The shape was scored with a scoring system according to Zeiger [33]. This system uses a 4-point scale (1 = single lumps of feces; 2 = shaped, with strong constrictions at the fecal surface; 3 = shaped with fissures at the fecal surface; 4 = shapeless). Fecal pH was determined before the collection phase at day 9 and on the last day of collection phase at day 14 by a digital pH meter (Mettler-Toledo GmbH, Gießen, Germany) placed in a fecal solution with distilled water (1 g/4 mL).

### 2.7. Statistical Analysis

Mean values as well as the standard deviation (SD) of the mean were calculated for all parameters. All recorded or measured parameters were analyzed individually and formed the basis of the calculation. To test for the normal distribution, a Shapiro–Wilk test was performed. For apparent tract digestibility in all groups and the food intake in both trials of the five-bowl tests, normal distribution could not be confirmed. To check the data for significant differences, a Kruskal–Wallis test and a pairwise Wilcoxon test were used. A simple ANOVA with a Tukey test afterwards was performed on the data that were normally distributed to determine the significant differences. All statements of statistical significance were based on *p* < 0.05. The calculations were performed using the statistical program R (v4.3.0) [34].

## 3. Results

### 3.1. Palatability Testing

The amount of food intake was significantly higher for the pumpkin press cake in the first run of the acceptance test (Table 5). In addition to that, the first choice was significantly more often the pumpkin press cake (Table 5).

In the second run of the acceptance test where the dogs received press cake with increasing proportions of potato flakes, there were no significant differences between the amounts of food between the different press cakes (Table 6).

### 3.2. Apparent Digestibility

None of the dogs refused food during the trials; all four diets were eaten completely. The apparent digestibility for organic matter was significantly higher for the pumpkin diet, reaching 90.11%, compared with the other diets. For crude protein, the apparent digestibility was significantly the lowest for the linseed diet (77.52%) and significantly the highest for the pumpkin diet (85.11%). For crude fat, the apparent digestibility was significantly higher for the pumpkin- and soy diet. The digestibility of the gross energy was significantly the lowest for the linseed diet (84.53%) and the sunflower diet (85.73%), while the other two diets had values over 88% and were comparable to one another (Table 7).

The digestibility of the amino acids did not differ significantly except for cysteine and tryptophan. The pumpkin and sunflower diets tended to yield slightly higher digestibility coefficients compared to soy and linseed diets for most amino acids. Among the essential amino acids, arginine and methionine exhibited the highest apparent digestibility values across all diets, whereas lysine and tryptophan showed comparatively lower and more variable results. The pumpkin-based diet had significantly the highest cysteine digestibility at 87.70%, while the soy-based diet had the lowest cysteine digestibility at 82.94%. Tryptophan digestibility, on the other hand, showed significant differences. The pumpkin-based diet had with 86.32% the highest tryptophan digestibility, while the linseed-based diet had with 76.33% the lowest digestibility (Table 8).

### 3.3. Fecal Quality

The defecation frequency was comparable between all diets as well as the scores for fecal consistency and fecal shape. The mass of fresh wet feces was significantly the highest for the linseed diet at 520.00 g over five days, while the fecal mass was 347.43 g over five days for the pumpkin diet, which covered 66.80% of the fecal mass of the linseed diet. The amount of feces deposited varied during the five-day collection phase. The lowest individual amount of feces was 14.5 g and the highest 196.9 g per day. The fecal dry matter content was not significantly different between all four diets. The pH values before the collection phase were 6.73 on average, whereas the pH values after the collection phase were 7.66. Within the two time points, the pH values for the different diets were comparable (Table 9).

## 4. Discussion

The palatability of five different oil mill-by-products was tested with two trials of a five-bowl test during the present study. The intake of the pumpkin press cake (34.47 g) in the first run was significantly the highest; besides that the first choice for the pumpkin press cake (4.8) was significantly the highest, too. Palatability can be affected by several different factors, like odor, food texture, flavor, and intrinsic variables of the dogs [35]. The typical roasty and nutty aroma from the pumpkin oil originates from roasting the seeds up to 130 °C [36]. During the roasting process, serval flavor compounds, like lipid peroxidation and Maillard reaction, are formed and are responsible for the aroma [37]. In the first run, the intake of rapeseed press cake was the lowest, just as it was chosen the least in the first choice. One possible explanation for this is the bitter substances in rapeseed and rapeseed proteins, which are a well-known phenomenon from human studies and can be traced back to kaempferol-based glycosides [38]. Although canine taste perception differs from that of humans, such findings may allow cautious inferences about possible aversive taste characteristics. In this study, a uniform consistency was achieved by adding water, thus minimizing the influence of food texture. In the second run, the overall intake of the rations was clearly higher and there were no significant differences between the different press cakes. This suggests that the potato flakes used had a decisive positive influence on the palatability. Potatoes contain almost all taste components that human taste receptors monitor: bitter, sour, sweet, and umami and starch can interact with the flavor components by influencing the texture [39]. Due to these properties of potatoes, the addition of potato flakes can also improve the flavor of gluten-free bread [40].

The effects of plant-based protein sources in a dog’s diets were analyzed on apparent nutrient digestibility and fecal characteristics during the present study. The digestibility of a diet can be affected by multiple factors, including the source and quality of the protein sources, the presence and type of fiber, the preparation and processing of the feed, and individual differences in the digestive system of dogs [41]. In our study, the organic matter digestibility was comparable for the sunflower- and linseed-diet and for the pumpkin- and soy diet; the two groups were significantly different from one another. The digestibility of organic matter for the soy diet was 87.30%, which is higher than findings from other studies, where the digestibility for a diet containing raw soybeans was 85.00%. Similarly, the organic matter digestibility of the pumpkin diet was 90.11%, exceeding results from previous studies [42,43]. Compared to the organic matter digestibility of green lentils (85.8%, literature data), the soy-based diet tested in the present study (87.30%) showed a higher digestibility. The pumpkin-based diet (90.11%) also exhibited higher organic matter digestibility than green lentils and reached a value comparable to that reported for peanut flour (90.30%). Literature data on pumpkin-based diets are limited; however, the high digestibility observed in the present study indicates efficient utilization of dietary components [44]. Even when compared to meat-based protein sources, the plant-based protein sources investigated in this study exhibited comparable or even superior organic matter digestibility. For instance, a chicken meal based diet in a previous study demonstrated an organic matter digestibility of 81.30%, which is lower than that observed for the soy (87.30%)- and pumpkin (90.11%) diet in the present study [45].

All diets fulfilled the recommendations for crude protein content for adult dogs [30]. In addition, all diets, except the linseed-diet, had protein digestibility comparable or higher than the recommended digestibility (80.00%) which is described by FEDIAF [30]. Furthermore, the protein digestibility of those three diets (sunflower-diet 84.23%, pumpkin-diet 85.11%, and soy-diet 82.35%) were comparable to the digestibility of another plant-based diet with pea protein (85.00%) in a previous study [46]. In earlier studies on plant-based protein sources in dog nutrition, the apparent digestibility of soy was the main focus of investigation. The results of the apparent digestibility of soy protein obtained in our study (82.35%) are comparable to those of previous studies (80.60–86.01%), and the protein digestibility of the sunflower- (84.23%) and pumpkin- (85.11%) diet are comparable to that of the soy diet [42,43,44]. Similarly, the protein digestibility of the plant-based protein sources evaluated in this study was comparable or higher than that of animal-based protein sources reported in previous studies. For example, a previous study reported protein digestibility values of 80.30% for poultry meal and 79.00% for salmon protein [47]. In comparison, all diets assessed in the present study, except for the linseed diet, exhibited higher protein digestibility. In this study, the sunflower- and linseed-diet had significantly lower apparent fat digestibility than the pumpkin- and soy diet. The fat digestibility for the sunflower diet (88.36%) and linseed diet (89.25%) are comparable to a diet with 30% of whole soya beans (89.98%) in a previous study [43]. All diets contained linseed oil as a fat source. However, the press cakes which were used contained residual amounts of fat. The added oil was also used to make the rations isoenergetic, which resulted in slight variations in the fat content across the different diets. The sunflower- and linseed diets had a higher fat content (103.99 g/kg DM and 102.25 g/kg DM, respectively) but were also higher in fiber content than the soy- and pumpkin-based diets. The higher fiber content in the sunflower- and linseed diets could be a reason for the lower protein and fat digestibility, as it could be shown that a high fiber content can have a negative effect on protein and fat digestibility [48]. The proportion of insoluble fiber in the linseed diet was the highest at 12.43%, and the digestibility determined for organic matter and crude protein was also significantly the lowest for this diet. It has been previously shown and is very well known that the type of fiber has an influence on digestibility, so a high proportion of insoluble fiber has a negative influence on organic matter and crude fiber digestibility [49]. Nevertheless, another reason could be that fecal fat concentration can be partially attributed to the bacteria in the colon, epithelia debris, and the de novo synthesis of fatty acids by the microbial population [50]. The high standard deviations in the apparent digestibility of fiber can be attributed to the fact that the analytical methods analyze different substances, in some cases also microbial polysaccharide compounds and ingested hair in different proportions [51]. As microbial activity was not directly measured in this study, the contribution of microbial polysaccharides to these variations has not been directly validated.

The apparent digestibility of the first limiting amino acids, methionine and lysine, was between 86.90% and 91.89% for methionine and between 78.38% and 85.15% for lysine. These results are largely comparable or higher than in previous studies, with five different dry meat-based canine foods, where the least square mean of the total tract digestibility was 82.60% for methionine and 76.80% for lysine [52]. In another study where different meat- or fish-based diets were used, the least square mean of the total tract digestibility was 82.00% for methionine and 78.90% for lysine, which is comparable with the study mentioned above and comparable or lower than the results in the present study [53]. In our study the sunflower diet had the highest methionine digestibility (91.89%) and the highest lysine digestibility (85.15%). The differences in the digestibility of the individual amino acids can be explained by the differences in the digestibility of the crude protein content. The sunflower and pumpkin diets had significantly higher crude protein digestibility compared to the other two diets. This is also reflected in the digestibility of the amino acids lysine and methionine. The methionine content of the sunflower diet (6.14 g/kg DM) was also higher than that of the pumpkin diet (5.44 g/kg DM), which also had a positive influence on the digestibility of methionine. Plant-based protein sources in canine diets can display imbalanced amino acid profiles, with methionine and lysine being the first limiting amino acids [22]. Insufficient supply of these amino acids can reduce protein utilization efficiency and compromise metabolic functions including taurine synthesis [22,54]. Therefore, diet formulation should be based on digestible amino acid findings, and supplementation with synthetic methionine and lysine should be applied when the amino acid balance of plant based ingredients does not meet the recommendations. In the present study, the diets used fulfilled the FEDIAF recommendations for amino acid supply in dogs [30].

To determine the fecal quality, defecation frequency, fecal consistency, fecal shape, and fecal output were used as measurable parameters in this study. The defecation frequency between the groups fed the four different protein sources did not differ. This is consistent with the data from previous studies where the defecation frequency did not differ between a meat-based and a vegetarian diet [14]. In addition, the fecal consistency scoring did not differ between the diets in this study. All diets were very close to the optimal score value of 2. These results are in line with the findings of another study where the fecal consistency was even very close to the optimal score value regardless of whether the diet contained 0%, 10%, 20%, or 30% of whole soy beans [43]. There is a documented negative effect of high soybean levels in dog food for the fecal texture [55]. This could not be confirmed in our study because the soy diet had a fecal consistency score of 2.17 (optimal score value 2). The fecal shape in a previous study by Zeiger was closely linked to the fecal consistency here as well and the values were close to the optimal score value of 2 [33]. The wet fecal output was significantly the highest for the linseed diet (520 g/5 d) in this study compared to the pumpkin-based diet with the lowest wet fecal output (347.43 g/5 d) as well as the lowest dry matter content (25.98%). This indicates that in absolute terms not only was the total fecal mass lower compared to meat- and insect-based diets reported in previous studies, but the actual amount of excreted solids was also reduced due to the higher water content [56]. The amount of wet fecal output is dependent on many factors like food intake, nutrient digestibility, the physiological state of the animal, and the water-holding capacity of the dietary ingredients [57]. In addition, it was shown that the processing of the feed has an influence on the amount of the wet feces [58]. Fecal dry matter content was not influenced by different protein sources and none of the dogs showed any gastrointestinal disorders like vomiting or diarrhea. The fecal dry matter content was comparable to another study which used a vegetarian diet [59]. Compared to a study which used meat-based diets where the dry matter content was lower, the dogs in this study had a higher fecal consistency than the optimum [60]. The pH values before the collection period were comparable and ranged between 6.65 and 6.91, these being similar to those from a previous study which used a diet with raw soy beans and had pH values ranging from 6.38 to 6.60 [42]. The recommended range for fecal pH is between 6 and 7. The pH values after the collection period were between 7.43 and 7.81. The pH value depends on the nutrient composition; an increased proportion of protein in the diet can effect a higher pH value [61]. This could not be confirmed in this study, as all diets were formulated to be isonitrogenous.

## 5. Conclusions

This study demonstrates that oil mill by-products can be well accepted as protein sources for adult dogs, providing protein with apparent digestibility values mostly over 80%. The findings indicate that nutrient digestibility and acceptance can vary significantly depending on the protein source. When fed in isolation, pumpkin seed cake had by far the highest acceptance. However, when combined with potato flakes, the differences between the offered protein sources became less distinct. Diets based on pumpkin (protein 85.11%; fat 93.05%) and soy (protein 82.35%; fat 92.50%) exhibited higher nutrient digestibility than those based on sunflower (protein 84.23%; fat 88.36%) and linseed (protein 77.52%; fat 89.25%). These results point out the importance of considering the digestibility of different protein sources when formulating plant-based diets for dogs to ensure optimal nutrient absorption. Furthermore, it has been demonstrated that oil mill by-products are suitable as a protein source due to their acceptability and digestibility, making them a viable option for practical use. In conclusion, it would be beneficial to conduct further research in the field of plant-based protein sources to explore not only their practical use and long-term health impacts but also to refine diet formulations for improved digestibility and nutritional balance.

## Figures and Tables

**Table 1:** Dietary composition in the second palatability run with dogs, combining potato flakes and one of five oil mill press cakes (hemp, linseed, sunflower, rapeseed, pumpkin).

Bowl	Potato Flakes in g	Press Cake in g	Water in g	Total in g
1	20	0	80	100
2	18	2	80	100
3	16	4	80	100
4	14	6	80	100
5	12	8	80	100

**Table 2:** Chemical composition (in g/kg dry matter) of the press cakes used in the palatability testing.

	Hemp	Linseed	Sunflower	Rapeseed	Pumpkin
Crude protein	379.87	384.44	399.14	287.82	624.73
Crude fat	92.85	123.67	151.16	124.52	123.65
Crude fiber	274.32	77.11	129.64	147.46	28.06
Crude ash	66.19	59.48	71.60	67.29	96.45

**Table 3:** Composition of the dogs’ diets for the apparent digestibility testing.

Components (% as Fed)	Soy Based-Diet	Linseed Based-Diet	Pumpkin Based-Diet	Sunflower Based-Diet
Soy press cake	20.00			
Linseed press cake		20.00		
Pumpkin press cake			20.00	
Sunflower press cake				20.00
Potato protein	7.40	11.20	3.70	9.60
Potato flakes	66.10	62.80	71.30	63.90
Linseed oil	6.50	6.00	5.00	6.50
Complementary feed *	6.00	6.00	6.00	6.00

* Consisting of minerals, vitamins and amino acids.

**Table 4:** Analyzed chemical composition (in g/kg dry matter if not otherwise stated) of the diets.

	Soy-Based Diet	Linseed-Based Diet	Pumpkin-Based Diet	Sunflower-Based Diet
Crude protein	207.26	204.72	201.81	211.97
Crude fat	92.89	102.25	80.67	103.99
Crude fiber	28.26	30.01	19.01	42.34
Insoluble dietary fiber in %	9.9	12.43	10.4	9.6
Crude ash	30.95	29.92	37.70	31.00
ME (MJ/kg DM)	16.64	16.86	16.55	16.44
GE (MJ/kg DM)	20.20	20.40	19.70	20.50
Calcium	8.00	8.00	8.00	8.00
Phosphorus	5.99	7.10	8.84	7.62
Arginine	12.63	12.32	19.43	13.80
Histidine	4.54	4.09	4.19	4.66
Isoleucine	9.42	9.28	8.05	9.62
Leucine	17.09	16.29	14.88	17.47
Lysine	13.35	12.43	10.08	12.73
Methionine	4.98	6.06	5.44	6.14
Phenylalanine	11.11	10.93	10.22	11.54
Threonine	9.00	9.24	8.50	9.46
Tryptophan	2.68	3.33	3.49	3.04
Valine	11.00	11.36	10.17	11.76
Alanine	9.50	9.56	8.97	10.05
Aspartic acid	28.63	26.36	24.11	26.86
Cysteine	4.06	5.12	4.23	5.11
Glutamic acid	34.40	31.94	34.52	36.78
Glycine	9.50	10.57	10.31	11.11
Proline *	10.63	9.80	8.88	10.78
Serine	10.89	10.45	10.14	10.66
Tyrosine	8.54	8.06	7.17	8.46
Taurine	1.80	1.80	1.80	1.80

* Hydroxyproline was not detected in any sample, indicating a purely plant-based composition of the diet.

**Table 5:** Average intake in dry matter and number of first choices for five different isolated press cakes (*n* = 8 dogs), with a maximum possible intake of 50 g per feedstuff.

	Hemp Press Cake	Linseed Press Cake	Sunflower Press Cake	Rapeseed Press Cake	Pumpkin Press Cake	*p* Values
Intake of press cake in g	2.98 ± 10.19 ^a^	16.55 ± 18.14 ^b^	4.86 ± 11.93 ^a^	1.05 ± 3.95 ^a^	34.47 ± 16.78 ^c^	<0.0001
Number of first choices/5 d	0.80 ± 1.30 ^a^	1.40 ± 1.14 ^a^	0.40 ± 0.55 ^a^	0.20 ± 0.45 ^a^	4.80 ± 1.64 ^a^	0.0062

^abc^ Values within a row marked with different superscript letters differ significantly (*p* < 0.05).

**Table 6:** Amount of food (dry matter in g) for different press cakes (PC) with decreasing proportions of potato flakes (PF) (*n* = 8 dogs).

	Hemp Press Cake (in g)	Linseed Press Cake (in g)	Sunflower Press Cake (in g)	Rapeseed Press Cake (in g)	Pumpkin Press Cake (in g)	*p* Values
0 g PC and 20 g PF	14.99 ± 5.32	17.35 ± 4.17	18.23 ± 1.66	16.20 ± 6.08	16.39 ± 5.35	0.7487
2 g PC and 18 g PF	14.17 ± 6.65 ^c^	18.12 ± 1.65 ^a^	17.94 ± 2.38 ^b^	14.61 ± 6.42 ^c^	15.10 ± 5.73 ^c^	0.0440
4 g PC and 16 g PF	15.47 ± 3.81	16.38 ± 5.21	15.70 ± 3.99	11.41 ± 6.53	15.20 ± 5.37	0.2301
6 g PC and 14 g PF	15.79 ± 3.02	15.48 ± 5.60	14.42 ± 6.60	10.99 ± 5.96	13.59 ± 7.15	0.1552
8 g PC and 12 g PF	16.52 ± 3.40	15.48 ± 6.00	11.97 ± 7.23	12.40 ± 5.64	14.88 ± 7.13	0.1105

^abc^ Values within a row marked with different superscript letters differ significantly (*p* < 0.05).

**Table 7:** Apparent nutrient digestibility in % in dogs (*n* = 8) fed the sunflower-, linseed-, pumpkin-, and soy diet (mean ± SD).

Digestibility of	Soy-Based Diet	Linseed-Based Diet	Pumpkin-Based Diet	Sunflower-Based Diet	*p* Values
Dry matter	84.70 ± 2.79 ^ab^	80.80 ± 4.15 ^a^	87.53 ± 1.54 ^b^	82.05 ± 2.10 ^a^	0.0004
Organic matter	87.30 ± 2.21 ^b^	83.17 ± 3.62 ^a^	90.11 ± 1.25 ^c^	84.10 ± 1.84 ^ab^	0.0001
Crude protein	82.35 ± 3.37 ^ab^	77.52 ± 6.04 ^b^	85.11 ± 1.77 ^a^	84.23 ± 2.20 ^a^	0.0019
Crude fat	92.50 ± 2.15 ^bc^	89.25 ± 2.97 ^ab^	93.05 ± 1.23 ^c^	88.36 ± 1.54 ^a^	0.0003
Crude fiber	12.77 ± 15.60	17.35 ± 23.76	21.37 ± 11.16	7.79 ± 10.02	0.0806
Gross energy	88.29 ± 2.12 ^ab^	84.53 ± 3.49 ^a^	90.57 ± 1.22 ^b^	85.73 ± 1.61 ^a^	0.0002

^abc^ Values within a row marked with different superscript letters differ significantly (*p* < 0.05).

**Table 8:** Apparent digestibility of amino acids (in %) in dogs (*n* = 8) fed the sunflower-, linseed-, pumpkin-, and soy diet (mean ± SD).

Digestibility of	Soy-Based Diet	Linseed-Based Diet	Pumpkin-Based Diet	Sunflower-Based Diet	*p* Values
Arginine	89.76 ± 1.89	85.89 ± 3.25	94.29 ± 0.73	91.78 ± 1.06	0.692
Histidine	86.50 ± 2.44	79.55 ± 4.67	87.83 ± 1.56	88.37 ± 1.82	0.716
Isoleucine	84.21 ± 2.81	79.69 ± 4.50	85.39 ± 1.68	86.33 ± 2.15	0.916
Leucine	85.94 ± 2.35	81.79 ± 4.09	87.30 ± 1.65	87.89 ± 1.87	0.952
Lysine	82.74 ± 2.90	78.38 ± 4.97	82.13 ± 2.11	85.15 ± 2.26	0.584
Methionine	87.85 ± 2.96	86.90 ± 4.14	91.22 ± 1.65	91.89 ± 2.06	0.160
Phenylalanine	87.54 ± 2.20	83.95 ± 3.71	89.35 ± 1.34	89.33 ± 1.55	0.997
Threonine	84.29 ± 2.53	78.79 ± 4.50	86.12 ± 2.20	85.67 ± 2.70	0.637
Tryptophan	78.91 ± 4.97 ^bc^	76.33 ± 5.14 ^c^	86.32 ± 2.30 ^a^	82.91 ± 2.28 ^ab^	<0.0001
Valine	83.38 ± 2.78	79.73 ± 4.49	85.46 ± 1.66	85.93 ± 2.18	0.753
Alanine	78.52 ± 3.23	72.99 ± 5.85	81.34 ± 2.22	81.87 ± 2.92	0.835
Aspartic acid	88.55 ± 1.93	84.76 ± 3.57	89.30 ± 1.25	89.17 ± 1.44	0.561
Cysteine	82.94 ± 3.04 ^c^	83.83 ± 4.37 ^bc^	87.70 ± 1.76 ^ab^	88.56 ± 1.75 ^a^	0.0004
Glutamic acid	87.97 ± 2.09	83.93 ± 3.25	90.67 ± 1.02	90.46 ± 1.22	0.561
Glycine	81.61 ± 2.94	79.44 ± 4.37	85.23 ± 1.57	85.38 ± 2.08	0.364
Proline	87.70 ± 1.98	83.03 ± 3.23	88.48 ± 1.34	89.20 ± 1.55	0.858
Serine	86.30 ± 2.04	81.19 ± 4.19	87.91 ± 1.73	87.34 ± 1.80	0.546
Tyrosine	87.37 ± 2.31	82.67 ± 3.71	87.12 ± 1.98	89.56 ± 1.42	0.704

^abc^ Values within a row marked with different superscript letters differ significantly (*p* < 0.05).

**Table 9:** Fecal characteristics of dogs (*n* = 8) fed the experimental diets (mean ± SD).

Parameter	Soy-Based Diet	Linseed-Based Diet	Pumpkin-Based Diet	Sunflower-Based Diet	*p* Values
Defecation frequency/d	2.15 ± 0.48	2.32 ± 0.43	1.77 ± 0.49	2.12 ± 0.12	0.381
Fecal consistency	2.17 ± 0.22	2.16 ± 0.20	2.12 ± 0.22	2.15 ± 0.13	0.782
Fecal shape	2.11 ± 0.13	2.12 ± 0.20	2.06 ± 0.10	2.09 ± 0.11	0.810
Fecal output (wet), g/5 d	378.63 ± 36.41 ^bc^	520.00 ± 75.62 ^a^	347.43 ± 33.54 ^c^	432.78 ± 27.46 ^b^	0.006
Fecal dry matter content (%)	29.15 ± 6.59	26.26 ± 6.04	25.98 ± 3.93	29.95 ± 4.51	0.761
pH value day 9					
(before collection phase)	6.66 ± 0.32	6.65 ± 0.65	6.91 ± 0.35	6.71 ± 0.28	0.868
pH value day 14					
(after collection phase)	7.81 ± 0.27	7.43 ± 0.85	7.66 ± 0.26	7.75 ± 0.53	0.620

^abc^ Values within a row marked with different superscript letters differ significantly (*p* < 0.05).

## Data Availability

The raw data supporting the conclusions of this article will be made available by the authors on request.
